# Longitudinal Morphological and Functional Assessment of RGC Neurodegeneration After Optic Nerve Crush in Mouse

**DOI:** 10.3389/fncel.2020.00109

**Published:** 2020-04-29

**Authors:** Liang Li, Haoliang Huang, Fang Fang, Liang Liu, Yang Sun, Yang Hu

**Affiliations:** ^1^Department of Ophthalmology, Stanford University School of Medicine, Palo Alto, CA, United States; ^2^Department of Ophthalmology, The Second Xiangya Hospital, Central South University, Changsha, China

**Keywords:** neurodegeneration, RGC, ON, OCT, SLO, PERG

## Abstract

The mouse optic nerve crush (ONC) model has been widely used to study optic neuropathies and central nervous system (CNS) axon injury and repair. Previous histological studies of retinal ganglion cell (RGC) somata in retina and axons in ON demonstrate significant neurodegeneration after ONC, but longitudinal morphological and functional assessment of RGCs in living animals is lacking. It is essential to establish these assays to provide more clinically relevant information for early detection and monitoring the progression of CNS neurodegeneration. Here, we present *in vivo* data gathered by scanning laser ophthalmoscopy (SLO), optical coherence tomography (OCT), and pattern electroretinogram (PERG) at different time points after ONC in mouse eyes and corresponding histological quantification of the RGC somata and axons. Not surprisingly, direct visualization of RGCs by SLO fundus imaging correlated best with histological quantification of RGC somata and axons. Unexpectedly, OCT did not detect obvious retinal thinning until late time points (14 and 28-days post ONC) and instead detected significant retinal swelling at early time points (1–5 days post-ONC), indicating a characteristic initial retinal response to ON injury. PERG also demonstrated an early RGC functional deficit in response to ONC, before significant RGC death, suggesting that it is highly sensitive to ONC. However, the limited progression of PERG deficits diminished its usefulness as a reliable indicator of RGC degeneration.

## Introduction

The retinal ganglion cell (RGC) is the only neuronal type to relay visual information from retina to the brain through the optic nerve (ON), which is formed by the projection axons sent exclusively from RGCs and conveniently separated from RGCs’ cell bodies in the inner retina. The unidirectional axon pathway in the ON is highly vulnerable to injury, which is the basis of a group of ON diseases called optic neuropathies. Progressive RGC death and ON degeneration are the common features of optic neuropathies, which are the leading cause of irreversible blindness (Levin, 1997; Burgoyne, 2011; Carelli et al., 2017; DeBusk and Moster, 2018). ON injury can be replicated definitively in a mouse by ON crush (ONC), which lesions all of the RGCs’ axons and leads to ON degeneration and retrograde RGC death (Minzenberg et al., 1995; Chierzi et al., 1999). Because of its reproducibility and precisely controlled injury site, ONC is widely used as a model of optic neuropathy (Park et al., 2008; Moore et al., 2009; Yang et al., 2014; Miao et al., 2016; Benowitz et al., 2017; Huang et al., 2017, 2019). Since optic neuropathy can also be associated with other central nervous systems (CNS) neurodegenerative diseases (Carelli et al., 2017), ONC provides a CNS neurodegeneration model that can be used for studying degenerative mechanisms and evaluating neuroprotectants and regeneration therapies.

The reason that glaucoma is called a “silent killer of vision” is that it is often diagnosed at a very late stage when neurodegeneration is already very severe. Thus, in the clinic, early detection of RGC/ON neurodegeneration and/or functional changes is critical to prevent irreversible loss of vision. Also, to evaluate neuroprotectants and regeneration therapies, we need to be able to follow RGC morphology and function longitudinally over a period to properly appreciate the effects of treatment. Postmortem histological study of mouse ONC has demonstrated severe degeneration of RGC somata in retina and axons in ON, but there has been no longitudinal morphological and functional assessment of RGCs in living animals. These unmet clinical needs motivate us to develop reliable *in vivo* assays in a well-controlled animal model to demonstrate practical and reliable ways to longitudinally assess RGC morphology and function. This capability will provide essential clinically relevant information for early detection and monitoring of the progression of CNS neurodegeneration.

Thinning of the retinal nerve fiber layer (RNFL) measured by optical coherence tomography (OCT) serves as a marker for optic neuropathies in the clinic (Costello et al., 2006; Balcer et al., 2015; Aktas et al., 2016), but the mouse RNFL is too thin to be reliably measured. Instead, the thickness of the retinal ganglion cell complex (GCC), including RNFL, ganglion cell layer (GCL) and inner plexiform layer (IPL), is often acquired by OCT to indicate the integrity of the RGC/ON in diverse optic neuropathy models in mouse (Nagata et al., 2009; Guo et al., 2010; Nakano et al., 2011; Kumar et al., 2019; Zhang et al., 2019a,b). Despite the usefulness of OCT traverse images of the retina, direct visualization of RGCs would be more informative. Although not yet feasible in the clinic, scanning laser ophthalmoscope (SLO) can readily image live transgenic mice with fluorescence-labeled RGCs (Leung et al., 2011; Chauhan et al., 2012; Smith and Chauhan, 2015). And the pattern electroretinogram (PERG) offers an electrophysiological assessment of RGCs in living animals (Chou and Porciatti, 2012; Chou et al., 2014; Porciatti, 2015).

Here, we report time course studies of RGC morphology and function after ONC by SLO, OCT, and PERG. Not surprisingly, direct visualization of RGCs by SLO fundus imaging is the procedure that correlates best with the histological quantification of RGC somata and axons. OCT finds retinal swelling in the first 5 days post-ONC and thinning at late time points. PERG is very sensitive to ONC, demonstrating a significant deficit 1-day post-ONC but fails to reveal progressive impairments after the initial response.

## Materials and Methods

### Mice

C57BL/6J WT mice were purchased from Jackson Laboratories (Bar Harbor, MI, USA). Thy1-YFP-17 transgenic mice were originally generated by Drs. Guoping Feng and Josh Sanes (Feng et al., 2000) and were acquired from Dr. Zhigang He (Sun et al., 2011; Belin et al., 2015). For all surgical and treatment comparisons, control and treatment groups were prepared together in single cohorts, and the experiment repeated at least twice. All experimental procedures were performed in compliance with animal protocols approved by the IACUC at Stanford University School of Medicine.

### ON Crush

ON crush was performed 2 weeks following AAV injection (Yang et al., 2014; Miao et al., 2016): the ON was exposed intraorbitally while care was taken not to damage the underlying ophthalmic artery and crushed with a jeweler’s forceps (Dumont #5; Fine Science Tools, Foster City, CA, USA) for 5 s approximately 0.5 mm behind the eyeball. Eye ointment containing neomycin (Akorn, Somerset, New Jersey, NJ, USA) was applied to protect the cornea after surgery.

### Immunohistochemistry of Whole-Mount Retina and RGC Counting

After transcardiac perfusion with 4% PFA in PBS, the eyes were removed, post-fixed with 4% PFA for 2 h, at room temperature, and cryoprotected in 30% sucrose overnight. Retinas were dissected out and washed extensively in PBS before blocking in staining buffer (10% normal goat serum and 2% Triton X-100 in PBS) for 30 min. RBPMS guinea pig antibody was custom made by ProSci Inc (Poway, CA, USA) and used at 1:4,000 as described before (Zhang et al., 2019b). Floating retinas were incubated with primary antibodies overnight at 4°C and washed three times for 30 min each with PBS. Secondary antibodies (Cy3) were then applied (1:200–400; Jackson ImmunoResearch, West Grove, PA, USA) and incubated for 1 h at room temperature. Retinas were again washed three times for 30 min each with PBS before a coverslip was attached with Fluoromount-G (SouthernBiotech, Birmingham, Alabama). RGC were counted, 6–9 fields randomly sampled from peripheral regions of each retina using a 40× lens and a Zeiss M2 epifluorescence microscope, and RBPMS^+^ RGCs were counted by Volocity software (Quorum Technologies Inc., Puslinch, ON, Canada). The percentage of RGC survival was calculated as the ratio of surviving RGC numbers in injured eyes compared to contralateral uninjured eyes. The investigators who counted the cells were blinded to the treatment of the samples.

### ON Semi-thin Sections and Quantification of Surviving Axons

The detailed procedure has been described previously (Zhang et al., 2019b). Briefly, transverse semi-thin (1 μm) sections of ON were cut on an ultramicrotome (EM UC7, Leica, Wetzlar, Germany) and collected 2 mm distal to the eye (about 1.5 mm distal to the crushed site). The semi-thin sections were stained with 1% para-phenylenediamine (PPD) in methanol: isopropanol (1:1). Four sections of each ON were imaged through a 100× lens of a Zeiss M2 epifluorescence microscope to cover the entire area of the ON without overlap. Two areas of 21.4 μm × 29.1 μm were cropped from the center of each image, and the surviving axons within the designated areas were counted manually. After counting all the images taken from a single nerve, the mean of the surviving axon number was calculated for each ON. The mean of the surviving axon number in the injured ON was compared to that in the contralateral control ON to yield a percentage of axon survival value. The investigators who counted the axons were masked to the treatment of the samples.

### Scanning Laser Ophthalmoscopy (SLO) Fundus Imaging and Quantification

After the pupil was fully dilated, the mouse retina was imaged by the SLO (Heidelberg Engineering GmbH, Heidelberg, Germany) at different time points under the same sensitivity (sensitivity 70), high-resolution mode (1,536 × 1,536 pixels) and 30 frames average with 480 nm excitation laser, 55° noncontact lens and the customized +10D contact lens (3.0 mm diameter, 1.6 mm BC, PMMA clear, Advanced Vision Technologies). The focal point position to the ON head, imaging area, and mouse position was fixed for every animal for reliable comparison. After ONC, the blue laser may cause the fully dilated pupil to contract during imaging, which will cause the fluorescence intensity to be lower than it actually is. Waiting a longer time to permit full pupil dilation will allow the best fundus fluorescence image to be acquired. The fluorescence intensity to radius measurement with the center at the optic disc was performed by ImageJ and the Concentric Circles Plugin as described (Wassmer et al., 2017). The summarized fluorescence intensity of each cycle to the radius was treated as an individual fluorescence intensity. The statistical analysis of the time course was based on the ratio of the ONC/naive eye.

### Spectral-Domain Optical Coherence Tomography (SD-OCT) Imaging

After the mice were anesthetized, pupils were dilated by applying 1% tropicamide sterile ophthalmic solution (Akorn, Somerset, NJ, USA), and the customized +10D contact lens applied to the dilated pupil. The retina fundus images were captured with the Heidelberg Spectralis SLO/OCT system (Heidelberg Engineering GmbH, Heidelberg, Germany) equipped with an 870 nm infrared wavelength light source and a 30° lens (Heidelberg Engineering). The OCT scanner has 7 μm optical axial resolution, 3.5 μm digital resolution, and 1.8 mm scan depth at a 40 kHz scan rate. The mouse retina was scanned with the ring scan mode centered by the ON head at 100 frames average under high-resolution mode (each B-scan consisted of 1536 A-scans). The GCC includes RNFL, GCL and IPL. The average thickness of GCC around the ON head was measured manually with the aid of Heidelberg software. The mean of the GCC thickness in the injured retina was compared to that in the contralateral control retina to yield a percentage of GCC thickness value. The investigators who measured the thickness of GCC were blinded to the treatment of the samples.

### Pattern Electroretinogram (PERG) Recording

Mice were anesthetized by xylazine and ketamine based on their body weight (0.01 mg xylazine/g + 0.08 mg ketamine/g). PERG recording of both eyes was performed at the same time with the Miami PERG system (Intelligent Hearing Systems, Miami, FL, USA) according to a published protocol (Chou et al., 2014). Briefly, mice were placed on a feedback-controlled heating pad (TCAT-2LV, Physitemp Instruments Inc., Clifton, NJ, USA) to maintain animal core temperature at 37°C. A small lubricant eye drop (Systane) was applied before recording to prevent corneal dryness. The reference electrode was placed subcutaneously on the back of the head between the two ears and the ground electrode was placed at the root of the tail. The active steel needle electrode was placed subcutaneously on the snout for the simultaneous acquisition of left and right eye responses. Two 14 cm × 14 cm LED-based stimulators were placed in front so that the center of each screen was 10 cm from each eye. The pattern remained at a contrast of 85% and a luminance of 800 cd/m^2^, and consisted of four cycles of black-gray elements, with a spatial frequency of 0.052 c/d. Upon stimulation, the independent PERG signals were recorded from the snout and simultaneously by asynchronous binocular acquisition. With each trace recording up to 1,020 ms, two consecutive recordings of 200 traces were averaged to achieve one readout. The first positive peak in the waveform was designated as P1 and the second negative peak as N2. P1 was typically around 100 ms. The amplitude was measured from P1 to N2. The mean of the P1-N2 amplitude in the injured eye was compared to that in the contralateral control eye to yield a percentage of amplitude change. The investigators who measured the amplitudes were blinded to the treatment of the samples.

### Statistical Analyses

GraphPad Prism 6 was used to generate graphs and for statistical analyses. Data are presented as means ± SEM. One-way ANOVA with a *post hoc* test was used for multiple comparisons.

## Results

### Postmortem Histology Shows Progressive RGC Death and ON Degeneration After ONC

**Figure 1:**
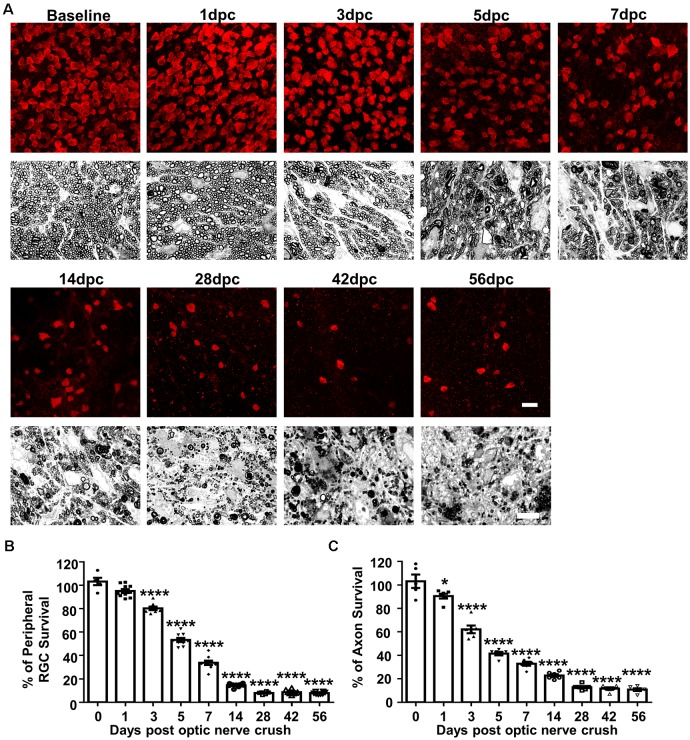
Histological study of progressive retinal ganglion cell (RGC) and ON degeneration after optic nerve crush (ONC). **(A)** Upper panel, confocal images of peripheral flat-mounted retinas showing surviving RBPMS positive (red) RGCs at different time points after ONC. Scale bar, 20 μm. Lower panel, light microscope images of semi-thin transverse sections of ON with para-phenylenediamine (PPD) staining. Scale bar, 10 μm. **(B,C)** Quantification of surviving RGCs (*n* = 6–10) and surviving axons in ON (*n* = 6) at different time points after ONC, represented as a percentage of ONC eyes compared to the contralateral naive eyes. Data are presented as means ± SEM *P < 0.05, ****P < 0.0001; one-way ANOVA with Tukey’s multiple comparisons test.

To determine the progression of RGC soma and axon degeneration after ONC, we first performed a postmortem histological time-course study. Eight groups of mature mice underwent ONC and were sacrificed at 1, 3, 5, 7, 14, 28, 42 or 56 days post crush (dpc). We counted RGC somata labeled with RBPMS in wholemount retinas and RGC axons labeled with PPD in semithin ON cross-sections (Figure 1A): compared to contralateral naïve eyes, about 95%, 80%, 53%, 34%, 15%, 8%, 9% and 8% RGCs survived and 91%, 62%, 42%, 33%, 23%, 13%, 12% and 11% ON axon survived at 1, 3, 5, 7, 14, 28, 42 and 56 dpc (Figures 1A–C). RGC axons exhibited minimal but significant loss at 1 dpc, whereas RGC somata numbers at 1 dpc did not significantly differ from those in contralateral naïve eyes, indicating that the axon degeneration preceded soma degeneration. No significant RGC soma or axon degeneration occurred after 28 dpc. We can only confidently quantify RGCs in the peripheral retina, where RGC density is significantly lower than in central retina, although the peripheral RGC survival should represent the whole retina. In contrast, when quantifying ON cross-section, we examined the whole area of ON. In summary, ONC induces progressive RGC and axon loss in the first 4 weeks and no additional degeneration thereafter. Thus we focus on the first 28 days post crush in the following experiments.

### *In vivo* Longitudinal SLO Retinal Imaging Shows Progressive RGC Loss, Correlating With Postmortem Histological Studies

**Figure 2:**
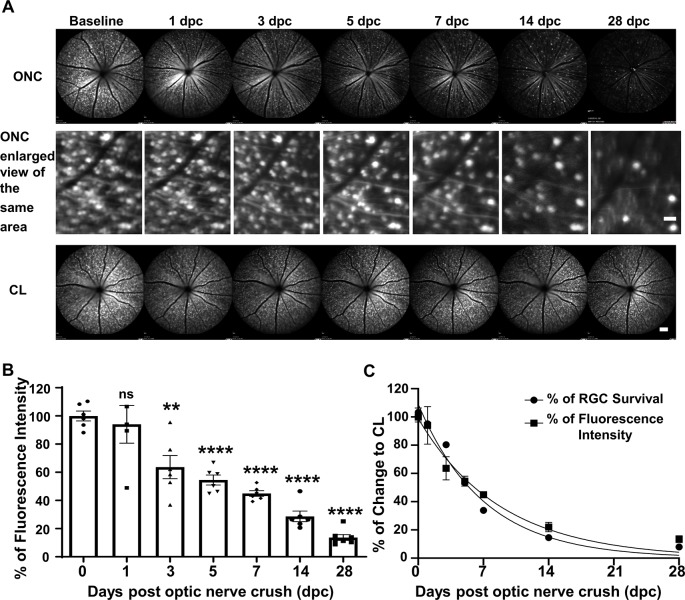
*In vivo* scanning laser ophthalmoscopy (SLO) fundus images showing progressive RGC lost after ONC. **(A)** Live SLO retina fundus images and enlarged SLO images of the same area showing YFP positive RGCs at different time points after ONC. Scale bar, 20 μm in the middle panel; 100 μm in the bottom panel. **(B)** Quantification of YFP fluorescence intensities at different time points after ONC, represented as a percentage of ONC eyes compared to the contralateral naive eyes (CL). Data are presented as means ± SEM, *n* = 5–7. ns: no significance, **p < 0.01, ****p < 0.0001, one-way ANOVA with Tukey’s multiple comparisons test. **(C)** The nonlinear regression decay curve fit of RGC fluorescence intensity acquired by SLO and RGC death quantification over the ONC time course from Figure 1B.

We used SLO to take retinal fluorescent fundus images of the same group of Thy1-YFP-17 mice, a transgenic mouse line labeling RGCs with YFP (Sun et al., 2011; Belin et al., 2015), at the same time points after ONC as the postmortem histological studies. SLO fundus images consistently demonstrated progressive total YFP signal loss, but ONC did not change the fluorescence intensities of the surviving RGCs (Figure 2A), indicating progressive RGC death. About 94%, 64%, 55%, 45%, 29% and 14% of RGCs survived at 1, 3, 5, 7, 14 and 28 dpc (Figures 2A,B). The decay curve based on fluorescence intensity was very similar to that of the RGC death curve acquired from the histological study (Figure 2C), confirming that SLO RGC imaging is a valid way to monitor RGC degeneration longitudinally.

### GCC Thickness as a Surrogate Marker for RGC Degeneration in Live Mice

**Figure 3:**
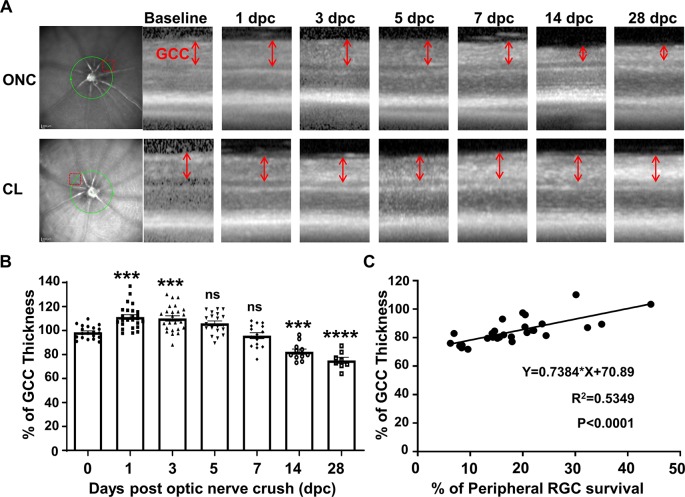
The dynamic change in ganglion cell complex (GCC) thickness detected by optical coherence tomography (OCT) in live mice after ONC. **(A)** Representative OCT images of mouse retina at different time points after ONC. The green circle indicates the OCT scan area surrounding the ON head; the red dashed box indicates the zoomed-in area shown in the right panel. GCC: ganglion cell complex, including retinal nerve fiber layer (RNFL), ganglion cell layer (GCL), and inner plexiform layer (IPL) layers; indicated as double-end arrows. **(B)** Quantification of GCC thickness, represented as a percentage of GCC thickness in the ONC eyes, compared to the contralateral naive eyes (CL). *n* = 8–25. Data are presented as means ± SEM, ns: no significance, ***p < 0.001, ****p < 0.0001, one-way ANOVA with Tukey’s multiple comparisons test. **(C)** Correlation analysis of RGC survival and GCC thickness at 14 and 28 dpc.

GCC thickness measured by OCT is often used as a morphological readout of dynamic RGC changes (Nakano et al., 2011; Zhang et al., 2019b). To monitor the progression of neurodegeneration *in vivo*, the changes of GCC thickness were acquired at different time points after ONC: Interestingly, the initial response of the retina to ONC was an increase in GCC thickness to 110% at 1 dpc and 3 dpc followed by a return to normal thickness at 5 dpc and 7 dpc (Figures 3A,B). Significant decreases of GCC thickness were only detected at 14 dpc (82%) and 28 dpc (75%; Figures 3A,B). Early retinal swelling in response to ONC may mask degeneration and compromise the usefulness of GCC thickness as a readout for RGC degeneration. However, the correlation between GCC thickness and RGC survival was significant at 14 and 28 dpc (Figure 3C), suggesting that GCC thickness monitored by OCT is a valid biomarker for late stages of RGC degeneration.

### PERG Is Sensitive to ONC Initially but Unresponsive to Further RGC Degeneration Until the Late Stages

**Figure 4:**
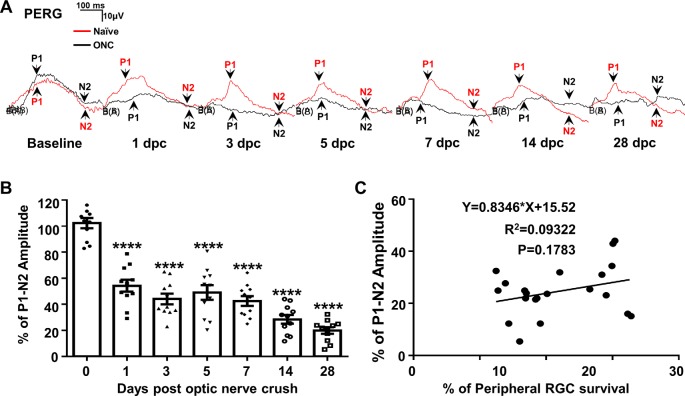
PERG is insensitive to RGC death in live animals after ONC. **(A)** Representative waveforms of PERG in the contralateral naive eyes (Naive, red lines) and the ONC eyes (ONC, black lines) at different time points after ONC. P1: the first positive peak after the pattern stimulus; N2: the second negative peak after the pattern stimulus. **(B)** Quantification of P1-N2 amplitude, represented as percentage of P1-N2 amplitude in the ONC eyes, compared to the contralateral naive eyes. *n* = 11. Data are presented as means ± SEM, ****p < 0.0001, one-way ANOVA with Tukey’s multiple comparisons test. **(C)** Correlation analysis of RGC survival and PERG amplitude at 14 and 28 dpc.

We measure PERG at the same time in both eyes (Chou et al., 2014) to normalize the injured eye with the contralateral control eye and minimize the variation inherent in electrophysiological recordings in live animals. The P1-N2 amplitude of the PERG decreased dramatically to 54% at 1 dpc (Figures 4A,B), indicating that PERG is very sensitive to ONC injury. However, the P1-N2 amplitude remained stable thereafter at 3, 5, and 7 dpc, and did not decrease further until a very late stage, when it became 28% at 14 dpc and 20% at 28 dpc (Figure 4B). The correlation between P1-N2 amplitude and RGC survival was very poor (Figure 4C).

## Discussion

### Direct RGC Visualization and GCC Thickness Are Reliable Biomarkers of RGC Neurodegeneration

Consistent with previous studies (Leung et al., 2011; Chauhan et al., 2012; Smith and Chauhan, 2015), we found that the retinal fundus imaging acquired by SLO can visualize and quantify the fluorescence-labeled RGCs directly; the quantification correlates highly with RGC death after ONC. Unfortunately, there is still no specific, safe way to fluorescently label RGCs in humans, which hinders the clinical application of this biomarker. Advancing imaging techniques to allow RGC visualization without fluorescent labeling or developing a non-toxic way to precisely label RGCs with fluorescence would help to solve this issue. RNFL thinning has been used in the clinic to indicate neurodegeneration in optic neuropathies. The GCC thinning that we observed at later time points after ONC reflects RGC degeneration and can serve as a biomarker for the ON injury-induced neurodegeneration. The dynamic changes in GCC thickness that we found in mouse retina after ONC, especially the early retinal swelling, are characteristic of diverse RGC/ON injuries; early GCC thickening has also been reported in mouse eyes after NMDA-induced RGC damage (Ohno et al., 2013) and in both mouse and human eyes with anterior ischemic optic neuropathy (Ho et al., 2013; Kupersmith et al., 2016). Monitoring these dynamic changes may provide a valuable biomarker for determining the retinal response to ON insult and neural repair treatment.

### The Need for a Reliable Functional Readout of RGC Status After ON Injury

PERG has been demonstrated to provide a useful electrophysiological assessment of RGC function in experimental animals (Chou and Porciatti, 2012; Chou et al., 2014; Porciatti, 2015) and has been applied to evaluate the effect of treatment in patients with optic neuropathy (Feuer et al., 2016; Guy et al., 2017). We therefore used PERG to monitor RGC function longitudinally after ONC. However, PERG deficits did not correlate with the progression of RGC/ON degeneration. Although there is no RGC death at 1 dpc, the P1-N2 amplitude of PERG has already decreased to 54%. A similar PERG response to ONC has been reported before (Chou et al., 2013), which suggests that a normal PERG requires uninterrupted retrograde ON signaling. We also found that the decreased PERG amplitude remained unchanged for at least 7 days after ONC, despite significant RGC death during this period. Therefore, our results indicate that the PERG is not a useful indicator of RGC degeneration and that it cannot serve to monitor RGC function longitudinally after ON injury or diseases. A more reliable, sensitive and non-invasive RGC functional readout is still needed to assess RGC status *in vivo*.

In summary, our study suggests that direct visualization of RGCs and retinal thickness are useful biomarkers for longitudinally monitoring neurodegeneration in optic neuropathies, whereas the challenge remains to develop a better RGC functional readout than PERG.

## Data Availability Statement

The datasets generated for this study are available on request to the corresponding author.

## Ethics Statement

The animal study was reviewed and approved by Institutional Animal Care and Use Committee (IACUC), Stanford University.

## Author Contributions

YH, LLi, and HH designed the experiments. LLi, HH, LLiu, and FF performed the experiments and analyzed the data. YH, LLi, HH, and YS prepared the manuscript.

## Conflict of Interest

The authors declare that the research was conducted in the absence of any commercial or financial relationships that could be construed as a potential conflict of interest.
